# Effectiveness and implementation of a MUltidisciplinary Lifestyle focused approach in the Treatment of Inpatients with mental illness (MULTI+): a stepped wedge study protocol

**DOI:** 10.1192/j.eurpsy.2022.2250

**Published:** 2022-09-01

**Authors:** M. Van Schothorst, N. Den Bleijker, I. Hendriksen, W. Cahn, N. De Vries, P. Van Harten, J. Deenik

**Affiliations:** 1 GGz Centraal, Scientific Research Department, Amersfoort, Netherlands; 2 LivIng Active, -, Santpoort-Zuid, Netherlands; 3 University Medical Centre Utrecht, Psychiatry, Utrecht, Netherlands; 4 Maastricht University, School For Mental Health And Neuroscience, Maastricht, Netherlands

**Keywords:** Health outcomes, Implementation, mental illness, protocol

## Abstract

**Introduction:**

People with mental illness (MI) have a reduced life expectancy compared to the general population, mostly attributable to somatic diseases caused by poor physical health. Modifiable lifestyle factors are increasingly associated with the onset of somatic diseases in people with MI. Despite the increasing evidence for the efficacy of lifestyle interventions there is little change in routine clinical care. This discrepancy is referred to as the implementation gap and has caused a need for effectiveness and implementation research in real-world settings.

**Objectives:**

This study investigates the health outcomes and implementation of a multidisciplinary lifestyle focused approach in treatment of inpatients with mental illness (MULTI+).

**Methods:**

This is an open cohort stepped wedge cluster randomized trial in inpatients psychiatric wards of GGz Centraal. Three clusters are randomly allocated to one of the three pre-defined steps to integrate MULTI+. MULTI+ can be tailored to fit individual psychiatric wards and includes 10 core components aimed at improving lifestyle factors. The primary outcome is to investigate whether there is a greater decrease in the QRISK3 cardiovascular risk score after receiving MULTI+ as compared to treatment as usual. Secondary outcomes include somatic and mental health outcomes, lifestyle factors, and implementation factors.

**Results:**

First results expected in 2022.

**Conclusions:**

To our knowledge, this will be the first large-scale study evaluating the long-term effects of a multidisciplinary, multicomponent approach aimed at improving lifestyle factors. We expect that this approach will increase long-term sustainability and can serve as a potential blueprint for future implementation of lifestyle interventions to improve routine clinical care.

**Disclosure:**

No significant relationships.

